# Longitudinal Ultrasound Evaluation of Cervical Length for Predicting Spontaneous Preterm Delivery Before 34 Weeks in Twin Gestations: A Retrospective Cohort Study

**DOI:** 10.3390/jcm15124523

**Published:** 2026-06-11

**Authors:** Takafumi Morinaga, Kazuma Onishi, Hiroyuki Tsuda, Yumiko Ito, Atsuko Tezuka, Tomoko Ando

**Affiliations:** 1 Department of Obstetrics and Gynecology, Japanese Red Cross Aichi Medical Center, Nagoya Daiichi Hospital, Nagoya 453-8511, Japan; takafumi0530mori@gmail.com (T.M.);; 2 Department of Obstetrics and Gynecology, Asada Ladies Clinic, Nagoya 450-0002, Japan

**Keywords:** cervical length, preterm birth, sequential evaluation, twin pregnancy, ultrasound

## Abstract

**Background/Objectives**: This study evaluated whether sequential changes in cervical length (CL) can predict spontaneous preterm birth (sPTB) before 34 weeks of gestation in twin pregnancies. **Methods**: This retrospective study from a single tertiary-care center analyzed 349 twin pregnancies with deliveries between January 2019 and December 2023. Cervical length assessments began at 18–21 weeks, followed by biweekly serial measurements. The primary outcome was sPTB before 34 weeks. CL changes were assessed descriptively using data from patients with and without sPTB before 34 weeks. We defined the high-risk status for sPTB based on our assessment. Logistic regression models were used to compute the odds ratios (ORs) and 95% confidence intervals (CIs) to quantify the relationship between these predictors and sPTB. Diagnostic accuracy was assessed from the area under the curve using receiver operating characteristic curve analysis. **Results**: The sPTB rate before 34 weeks of gestation was 8.5% (18/212). In the group without -sPTB before 34 weeks of gestation, the 5th percentile CL was approximately 20 mm at 25 weeks and 15 mm at 26–27 weeks of gestation. Sequential CL measurements revealed that a rapid shortening of ≥10 mm within 2 weeks significantly predicted sPTB before 34 weeks. A decrease in CL of ≥10 mm in a 2-week interval was associated with increased odds for sPTB before 34 weeks of gestation [adjusted OR (95% CI): 6.66 (2.32–19.14)]. **Conclusions**: In twin pregnancies, measuring CL every 2 weeks after approximately 20 weeks of gestation may facilitate sPTB detection before 34 weeks.

## 1. Introduction

Twin pregnancies carry a higher risk of complications than singleton pregnancies. Preterm delivery is the leading contributor to perinatal mortality, neonatal morbidity, and long-term complications in twins [1]. In the USA, 10% of live births in 2022 were preterm (<37 weeks), including 3% before 34 weeks; conversely, in twin pregnancies in 2022, these rates were 60% and 20% respectively [2]. In Japan, the 2021 Vital Statistics reported preterm birth rates of 4.7% in singletons and 51.9% in multiples, with 20.3% occurring before 34 weeks of gestation. The accurate prediction of preterm birth, particularly before 34 weeks of gestation, is therefore crucial in twin pregnancies. Cervical length (CL) is a reliable predictor of preterm birth in twin pregnancy, and 25 mm is a pragmatic cutoff for 18–24 weeks of gestation [3]. A CL of <20 mm at 20–24 weeks of gestation optimally predicts preterm birth before 34 weeks in asymptomatic twin pregnancies [4]. According to a meta-analysis, CL < 30 mm at 18 weeks best predicts preterm birth before 28 weeks, whereas CL measured later in pregnancy was more useful for predicting preterm birth after 28 weeks, prompting recommendations to measure CL at 18 and 22 weeks [5]. Some studies reported that four CL measurements between 18 and 32 weeks of gestation further improve predictive accuracy [6,7]. While CL screening is common during 16–24 weeks in many countries, the current Japanese Society of Obstetrics and Gynecology (JSOG) guidelines (2023) do not recommend routine CL measurements in asymptomatic cases.

As noted previously, there is ongoing debate regarding the optimal threshold and timing for measuring CL in twin pregnancies, and no consensus has been reached. Moreover, although mid-trimester CL assessment is considered important, evidence supporting the usefulness of measuring CL after 24 weeks of gestation or evaluating the degree of cervical shortening over time remains inconclusive. Therefore, this study aimed to exploratively investigate the longitudinal trajectory of CL in twin pregnancies. Specifically, we sought to determine whether temporal changes in CL are associated with an increased risk of spontaneous preterm birth before 34 weeks, thereby contributing to the potential development of future standardized surveillance strategies.

## 2. Materials and Methods

### 2.1. Subjects

This retrospective cohort study was conducted at a tertiary care facility between January 2019 and December 2023. Clinical and demographic parameters were collected from electronic medical and midwifery charts and included maternal age, chorionicity, gestational age at delivery, admission body mass index (BMI), gestational age at the time of CL measurement, CL, parity, previous preterm birth, hypertensive disorders of pregnancy (HDP), gestational diabetes mellitus (GDM)/diabetes mellitus (DM), twin-to-twin transfusion syndrome (TTTS) status, cervical cerclage, intrauterine fetal death (IUFD), major fetal anomalies, history of cervical surgery, uterine fibroids, and the utilization and type of assisted reproductive technology (ART). The inclusion criteria required participants to begin CL measurements between 18 weeks 0 days and 21 weeks 6 days of gestation, with subsequent follow-up every 2 weeks until delivery. The exclusion criteria for the analysis included patients with TTTS complications, those requiring cervical cerclage, IUFD, major congenital anomalies, follow-up initiation after 21 weeks and 6 days of gestation. In addition, since the objective of this study was to elucidate the causal relationship between changes in the CL and spontaneous preterm birth (sPTB), iatrogenic preterm birth before 34 weeks of gestation was excluded because it results in delivery regardless of the natural changes in the CL.

All individuals were examined by experienced obstetricians using transvaginal ultrasonography (Voluson S8 or E10; GE Healthcare, Tokyo, Japan). The CL was determined in the sagittal view, and the probe’s depth and angle were adjusted to provide a clear view of the complete cervical canal, extending from the internal to the external os [8]. Great care was taken to avoid cervical compression, and the image magnification, frequency, and gain settings were optimized for maximal contrast. The CL was measured two to three times, and the minimum value was recorded.

### 2.2. Management

The estimated due date was primarily based on the last menstrual period. However, ultrasound-based dating was prioritized if the crown-rump length measured between 8 and 10 weeks differed by >7 days. In cases of ART, the fertilization date was used as the definitive dating method. First-trimester ultrasonography was also performed to ascertain chorionicity and amnionicity [9]. CL was serially assessed starting from 18 weeks 0 days to 21 weeks 6 days of gestation, with subsequent measurements taken at least every 2 weeks. The shortest CL recorded within the specified 2-week intervals (18w0d–21w6d, 22w0d–23w6d, …, 32w0d–33w6d) was designated as the representative value for that period. Weekly surveillance was performed for patients with suspected cervical shortening. Vaginal progesterone and cervical pessaries were not used in the management protocols for these twin pregnancies. Therapeutic cerclage was an option for women presenting with suspected cervical insufficiency before 25 weeks’ gestation and CL ≤ 15 mm. Symptomatic patients experiencing regular painful contractions, hemorrhage, or signs of cervical dilation or effacement were hospitalized and treated as having a high risk for preterm birth. All associated prenatal and ultrasound data were recorded in medical charts.

### 2.3. Statistical Analyses

Clinical data from medical records were converted into digital format and input into a computerized spreadsheet program (Excel, Microsoft Japan Co., Ltd., Tokyo, Japan). The initial analyses focused on the demographic and clinical characteristics of the twin delivery cohort, specifically maternal age, BMI at admission, obstetric history, medical history, and chorionicity.

Further, this study aimed to investigate patterns of CL across gestational ages in women with and without sPTB before 34 weeks by performing descriptive analyses from two viewpoints: (1) CL at each gestational age and (2) change in CL over 2-week intervals. For individuals without sPTB before 34 weeks, the CL distribution was assessed prospectively from 18 to 21 and 32 to 33 weeks of gestation. For individuals with sPTB before 34 weeks of gestation, CL was assessed retrospectively by counting backward from the gestational age at delivery. Changes in CL over 2-week intervals were analyzed in the same manner. CL values and their changes were considered predictive factors for sPTB before 34 weeks. High-risk individuals were defined based on the outcomes of this descriptive assessment, which involved both absolute CL and changes in CL. Logistic regression models were used to compute the odds ratios (ORs) and 95% confidence intervals (CIs) to quantify the relationship between these predictors and sPTB. Maternal age was selected a priori and included in the adjusted analyses as a potential confounding variable. Diagnostic accuracy was assessed using receiver operating characteristic (ROC) curve analysis to determine the area under the curve (AUC). Furthermore, sensitivity, specificity, and positive predictive value (PPV) and negative predictive value (NPV) were derived. Missing CL measurements necessitated both a complete-case analysis and an analysis using multiple imputation for missing values to calculate the odds ratios. The descriptive analysis of CL utilized the full study population without imputation. Statistical tests were performed using a two-sided alpha value of 0.05, which was set as the threshold for statistical significance. Stata version 18.5 (StataCorp LLC, College Station, TX, USA) was used for all analyses.

## 3. Results

**Figure 1:**
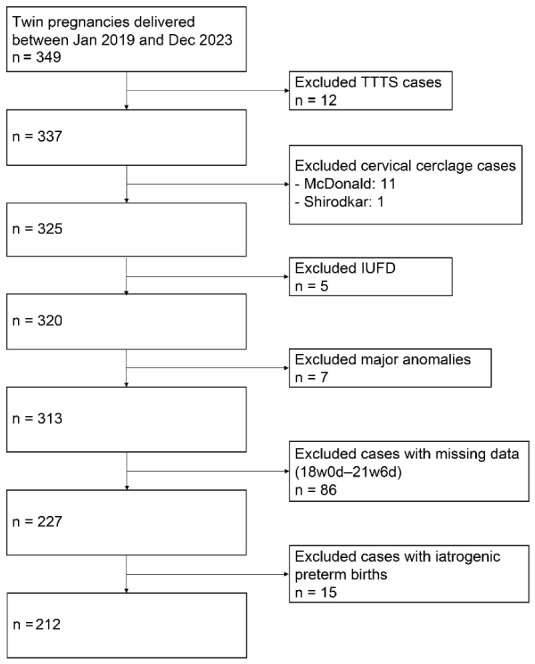
Recruitment Flowchart of Twin Pregnancies Eligible for Cervical Length Analysis.

In total, 349 twin pregnancies were delivered at our institution between January 2019 and December 2023. After excluding the patients who met the exclusion criteria, 212 twin pregnancies were included in the study (Figure 1). Patient backgrounds and characteristics are summarized in Table 1. No differences were found between the two groups regarding background factors, such as age, BMI, parity, rate of ART pregnancies, and chorionicity. In this study, the rate of spontaneous preterm births before 34 weeks of gestation was 8.5% (18/212).

Information regarding missing values is presented in Table S1. A total of 81 individuals had complete CL data, whereas 131 individuals had at least one missing CL value. Approximately 80% of patients with missing CL values had at most two missing values.

**Figure 2:**
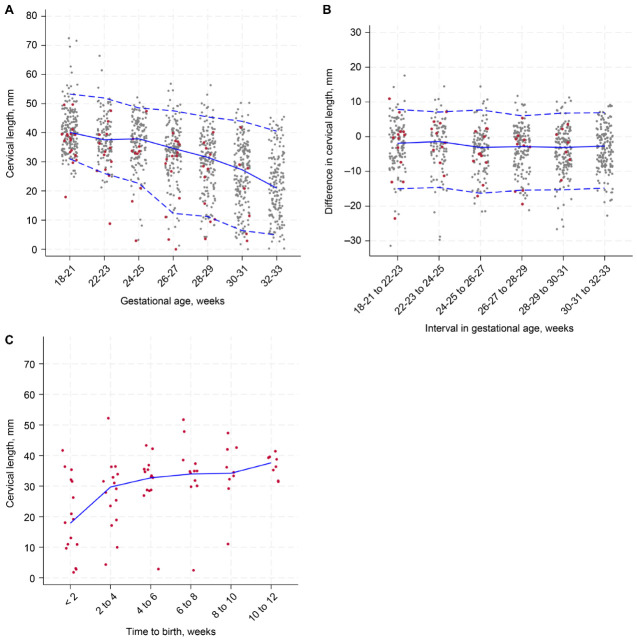
Association Between Cervical Length (CL) and Pregnancy Outcomes. (**A**). Distribution of CL measurements by gestational age. (**B**). Sequential changes (differences) in CL between 2-week intervals. For (**A**,**B**): Grey dots, cases without sPTB; Red dots, cases with sPTB; Solid blue lines, median values; Dashed blue lines, 5th and 95th percentiles for the non-sPTB group. (**C**). Retrospective changes in CL from the date of delivery in spontaneous preterm births (sPTB) before 34 weeks (N = 18). Red dots represent individual measurements, and the solid blue line indicates the median progression. (10–12 weeks, 37.6 mm; 8–10 weeks, 34.2 mm; 6–8 weeks, 34.0 mm; 4–6 weeks, 32.7 mm; 2–4 weeks, 29.7 mm; <2 weeks, 19.7 mm).

The distribution of CL in this study is shown in Figure 2A. The solid line is the median value for the non-sPTB group before 34 weeks of gestation, and the dashed lines are the 5th and 95th percentiles, respectively. The red dots represent the sPTB group at less than 34 weeks of gestation. CL gradually shortened as gestational age progressed. In addition, at the 5th percentile, CL was approximately 20 mm at 25 weeks of gestation and 15 mm at 26–27 weeks of gestation.

CL differences between 2-week intervals in this study are shown in Figure 2B. Regarding the change in CL every 2 weeks, consistent and gradual shortening was observed.

The retrospective changes in CL from the date of delivery to sPTB before 34 weeks of gestation are shown in Figure 2C. Regarding changes in CL every 2 weeks, although a consistent and gradual shortening was generally observed, a marked reduction of more than 10 mm in CL within 2 weeks before delivery was also noted (a median decrease from 29.7 mm to 17.9 mm; this was not statistically significant compared with the shortening observed 2 to 4 weeks before delivery).

Based on the descriptive analysis of CL in patients with and without sPTB before 34 weeks of gestation, we defined a high-risk group in three ways: (Model 1) those with CL ≤ 20 mm at 22w0d–25w6d or CL ≤ 15 mm at 26w0d–33w6d, or a decrease in CL of ≥10 mm in a measurement interval; (Model 2) those with CL ≤ 20 mm at 22w0d–25w6d or CL ≤ 15 mm at 26w0d–33w6d; (Model 3) those with a decrease in CL of ≥10 mm in a measurement interval.

For those patients categorized as high risk by Model 1, the adjusted odds ratio (aOR) for sPTB before 34 weeks of gestation compared with non-high-risk individuals was 6.77 (95% CI, 1.90–24.12) (Table 2).; furthermore, the AUC was 0.71 (95% CI, 0.61–0.82); sensitivity, 0.78; specificity, 0.63; PPV, 0.17; and NPV, 0.97. For those categorized as high risk by Model 2, the aOR for sPTB before 34 weeks of gestation compared with non-high-risk individuals was 3.84 (95% CI, 1.33–11.08) (Table 2); furthermore, the AUC was 0.65 (95% CI, 0.53–0.77); sensitivity, 0.50; specificity, 0.79; PPV, 0.18; and NPV, 0.94. For those categorized as high risk by Model 3, the aOR for sPTB before 34 weeks of gestation compared with non-high-risk individuals was 6.66 (95% CI, 2.32–19.14) (Table 2); furthermore, the AUC was 0.71 (95% CI, 0.59–0.83); sensitivity, 0.61; specificity, 0.81; PPV, 0.23; and NPV, 0.96. The results of the complete-case analysis were consistent with those of the analysis using multiple imputations, although the high-risk group in Model 2 was not significantly associated with increased sPTB in the complete-case analysis (Table S2).

## 4. Discussion

According to 2022 data from the USA, approximately 20% of twins were born before 34 weeks of pregnancy [2]. According to Japan’s 2021 Vital Statistics, the rate of preterm birth at <34 weeks of gestation was 20.3% for multiple pregnancies. Therefore, preterm birth is more common in twin pregnancies, resulting in higher morbidity and mortality rates. Twin pregnancy is an independent risk factor for preterm birth, possibly due to uterine overdistension [10]. Therefore, highly accurate indicators are needed to predict preterm birth (especially before 34 weeks of gestation) in twin pregnancies. The prognostic utility of CL remains unclear when applied to twin gestation. Thus, we conducted this study to propose new indicators by analyzing data obtained from biweekly CL measurements in the same patients.

Our hospital protocol mandates serial follow-up examinations—every 2 weeks—beginning at 16 weeks for monochorionic twins and 22 weeks for dichorionic twins. These assessments were conducted to evaluate fetal growth, amniotic fluid levels, and fetal blood flow. Additionally, we measured CL at approximately 20 weeks of gestation and then every 2 weeks from 22 weeks onwards. As far as we know, few reports have frequently measured CL in twin pregnancies and analyzed the resulting data, which we consider a strength of this study. Additionally, the use of consistent ultrasound equipment and a standardized measurement method contributed to the high quality of the data, which is another strength of this study.

Practice patterns regarding CL measurement in twin pregnancies vary among clinicians, given the lack of high-quality evidence favoring one approach over another [11]. A short cervix is generally defined as a cervix with CL < 20 mm or ≤25 mm in twin pregnancies. However, a specific CL threshold that can reliably confirm or dismiss the risk of spontaneous preterm delivery has not been definitively identified. Vaginal progesterone reduces preterm delivery before 33 weeks and improves neonatal outcomes in twin pregnancies with a second-trimester CL ≤ 25 mm [12]. It also lowers early preterm birth and neonatal morbidity/mortality in twin gestations where a short cervix (≤30 mm) was detected by ultrasonography [13]. Cerclage benefits twin pregnancies with CL < 15 mm by reducing preterm deliveries and prolonged gestation [14]. These findings underscore the importance of mid-trimester CL assessment. However, evidence supporting the usefulness of measuring CL after 24 weeks of gestation or evaluating the degree of cervical shortening over time remains inconclusive. Furthermore, the number of CL measurements varies among studies, with some reporting one [4], two [5], or four measurements [6,7]. Recently, a cost-utility analysis revealed that a universal screening strategy involving two rounds of screening at 18–20 weeks and 20–22 weeks was found to be most beneficial in a Canadian cohort of dichorionic diamniotic twin pregnancies with no history of preterm birth or prior prophylactic progesterone administration or cervical cerclage [15]. Numerous reports exist on the timing, number of measurements, and cutoff values, yet no consistent guidelines have been established [11]. Recently, a meta-analysis of individual participant data from twin pregnancies with available mid-trimester transvaginal ultrasound CL measurements reported a mean CL of 39 mm at 16–26 weeks [16]. The prognostic value of CL for predicting sPTB was linear, with the risk of sPTB at <34 weeks reduced by 6.8% for every millimeter increase in CL [17]. This finding is notable because the prognostic value of CL in predicting sPTB is linear; our results support this observation, showing a linear pattern in the non-sPTB group. Conversely, the rapid shortening of the CL just before preterm birth in the sPTB group (<34 weeks) is a characteristic and novel finding that has not been reported previously. While conventional screening methods rely on a static, single-point CL threshold to identify high-risk asymptomatic twin pregnancies, these single-point assessments often fail to capture rapid, acute cervical changes that occur between routine checkups. The clinical value of our proposed dynamic indicator (≥10 mm reduction within 2 weeks) lies in its ability to detect rapidly progressing cervical shortening even when the absolute CL value appears “safe.” This dynamic approach does not replace the traditional single-point threshold monitoring; rather, it serves as a complementary assistance tool. By combining static thresholds with sequential evaluation, clinicians can better identify the risk for sPTB before 34 weeks of gestation. Until now, CL has been measured every 2 weeks during the management of twin pregnancies. The motivation for conducting this study was that measuring the CL every 2 weeks places a considerable burden on both physicians and patients and may result in excessive medical intervention. Therefore, we considered whether reducing the number of CL measurements would be possible but ultimately recognized the importance of measuring biweekly assessments. Specifically, if the CL shortens by 10 mm or more within a 2-week interval, the risk of sPTB before 34 weeks of gestation is high, and delivery is imminent, necessitating close monitoring. On the other hand, extending the measurement interval beyond 2 weeks (e.g., to 3 or 4 weeks) may be clinically unsafe for twin pregnancies. Acute cervical shortening may progress rapidly within days. We believe that a biweekly interval is the maximum window for practically capturing these progressive changes. Extending the interval would increase the risk of missed diagnoses.

Our findings are consistent with those of previous studies demonstrating the prognostic value of serial CL measurements and the trajectory of cervical shortening in twin pregnancies [5,6,7]. However, an important clinical consideration in interpreting our results is the concept of reverse causality. The rapid >10 mm shortening observed shortly before delivery may not simply serve as an early predictive marker but may reflect the evolving physiological process of imminent preterm labor itself. While detecting this rapid change highlights a state of high risk, its modest overall predictive discrimination (AUC: 0.71) and low positive predictive value suggests that it identifies pregnancies already transitioning into active labor. Thus, implementing routine biweekly CL surveillance to capture this rapid change in CL must be carefully weighed against the increased resource utilization and patient burden, because doing so may not provide enough lead time to alter clinical outcomes significantly. Nevertheless, we believe that identifying this “sign of imminent labor onset” via biweekly surveillance still holds significant clinical utility. In the management of twin pregnancies, capturing this acute shortening allows clinicians a critical window—even if short—to initiate immediate interventions, such as hospitalization, administration of antenatal corticosteroids for fetal lung maturation, and transfer to a tertiary center.

A key strength of this study lies in its single-center design with a standardized protocol for biweekly transvaginal ultrasound assessments by experienced obstetricians, ensuring high-quality and consistent data collection. However, this study has inherent limitations. First, consistent with the existing literature [17], our institutional policy dictates delivery for dichorionic twins at 37 weeks and for monochorionic twins at 36 weeks of gestation. Therefore, preterm birth in this study was defined as delivery before 34 weeks rather than that before 37 weeks. Second, we excluded 86 patients whose follow-up started after 21 weeks and 6 days because they lacked CL data before follow-up, which could have introduced bias. Our hospital is a tertiary-care center, and because some cases were referred during the second half of the pregnancy, a proportion of patients did not meet the inclusion criteria. Third, we did not administer vaginal progesterone or insert pessaries in cases of cervical shortening. Implementing these treatments may help reduce preterm birth rates. Fourth, the number of endpoint events was relatively small, with only 18 cases of sPTB before 34 weeks included in our cohort. This small number of positive events inevitably limited the statistical power of our predictive models and logistic regression analyses, and we limited our multivariable adjustment to maternal age. Consequently, this constraint may increase the risk of type II errors and affect the precision of the estimated ORs and CIs. In addition, the cutoff (>10 mm reduction within 2 weeks) was derived from the descriptive analysis. Therefore, our findings should be interpreted as exploratory, and large-scale, multicenter studies are required to validate the statistical robustness of these dynamic thresholds. Fifth, we excluded twin pregnancies with iatrogenic preterm births as in previous studies. Interestingly, Hughes et al. [16] showed that CL exhibits similar prognostic performance for sPTB and all preterm deliveries including iatrogenic birth. Future studies should determine whether the CL remains unaffected by these conditions. Sixth, the minimum CL value was recorded as the CL analyzed in this study to eliminate bias caused by temporary changes in CL caused by uterine contractions and to assess the condition of highest risk in clinical practice. Furthermore, although measurements were performed by experienced obstetricians following standardized procedures, a rigorous quantitative assessment of intra-observer variability was not conducted because of the retrospective nature of the study. Lastly, missing data were present in the CL measurements collected every 2 weeks. Although data imputation is often associated with potential bias, comparative analysis revealed no statistically significant differences in key outcomes between the imputed and complete-case datasets. This finding suggests that the results are robust to the chosen method of handling missing data.

## 5. Conclusions

We demonstrated that, in twin pregnancies with sPTB before 34 weeks, the CL rapidly shortened by ≥10 mm within 2 weeks of delivery. Multivariate logistic analysis showed that a decrease in CL of ≥10 mm within a 2-week interval was associated with an aOR of 6.66 for the risk of sPTB before 34 weeks. In conclusion, sequential monitoring of CL from 20 weeks of gestation, specifically focusing on the velocity of cervical changes (e.g., reduction of ≥10 mm within 2 weeks), could serve as a valuable clinical monitoring strategy to identify pregnancies at imminent risk for sPTB before 34 weeks. While international consensus on routine twin CL screening remains controversial, our data support the utility of longitudinal, biweekly tracking to prevent missing acute, progressive cervical shortening. However, given the exploratory nature of these data-driven cutoffs, the modest predictive values, and the possibility that this rapid shortening reflects imminent labor rather than actionable early prediction, caution is warranted when applying these findings to future clinical practice. Future prospective, external validation studies are needed to determine the true clinical utility of these dynamic thresholds.

## Figures and Tables

**Table 1:** Comparison of baseline characteristics between the sPTB (<34 weeks) and non-sPTB (<34 weeks) groups.

Demographics	sPTB at <34 Weeks	Not sPTB at <34 Weeks	*p*-Value
	(*n* = 18)	(*n* = 194)	
Maternal age, mean (SD)	32.1 (5.3)	32.3 (4.6)	0.89
BMI on admission, median (25–75th percentile)	20.9 (18.9–23.0)	20.5 (18.9–22.4)	0.55
Parity			0.33
Nulliparous	12 (66.7)	105 (54.1)	
Multiparous	6 (33.3)	89 (45.9)	
Previous preterm birth	1 (5.6)	3 (1.5)	0.3
Medical history			
Conization	1 (5.6)	0 (0.0)	0.08
GDM/DM	1 (5.6)	6 (3.1)	0.47
HDP	2 (11.1)	19 (9.8)	0.69
Myoma	0 (0.0)	4 (2.1)	1.00
ART	7 (38.9)	54 (27.8)	0.41
Chorionicity			0.09
DD	13 (72.2%)	128 (66.0%)	
MD	4 (22.2%)	64 (33.0%)	
MM	1 (5.6%)	2 (1.0%)	

sPTB, spontaneous preterm birth; BMI, body mass index; GDM, gestational diabetes mellitus; DM, diabetes mellitus; HDP, hypertensive disorders of pregnancy; ART, assisted reproductive technology; DD, dichorionic diamniotic; MD, monochorionic diamniotic; MM, monochorionic monoamniotic.

**Table 2:** Odds ratios for spontaneous preterm delivery at <34 weeks of gestation calculated for each risk factor using multivariate analysis (after multiple imputation).

	sPTB at <34 Weeks (*n* = 18)	Not sPTB at <34 Weeks (*n* = 194)	Crude OR (95% CI)	Adjusted OR (95% CI)
Model 1 *				
High risk (*n* = 85) (%)	16.5 (14/85)	83.5 (71/85)	6.77 (1.90–24.09)	6.77 (1.90–24.12)
Non-high risk (*n* = 127) (%)	3.1 (4/127)	96.9 (123/127)	reference	reference
Model 2 †				
High risk (*n* = 50) (%)	18.0 (9/50)	82.0 (41/50)	3.83 (1.33–11.05)	3.84 (1.33–11.08)
Non-high risk (*n* = 162) (%)	5.6 (9/162)	94.4 (153/162)	reference	reference
Model 3 ‡				
High risk (*n* = 47) (%)	21.5 (11/47)	78.5 (36/47)	6.57 (2.30–18.79)	6.66 (2.32–19.14)
Non-high risk (*n* = 165) (%)	4.2 (7/165)	95.8 (158/165)	reference	reference

sPTB, spontaneous preterm birth; OR, odds ratio; CI, confidence interval. *: High risk was defined as cervical length ≤ 20 mm at 22w0d–25w6d or cervical length ≤ 15 mm at 26w0d–33w6d, or a decrease in cervical length ≥ 10 mm in a measurement interval. †: High risk was defined as cervical length ≤ 20 mm at 22w0d–25w6d or cervical length ≤ 15 mm at 26w0d–33w6d. ‡: High risk was defined as a decrease in cervical length ≥ 10 mm in a measurement interval.

## Data Availability

The data presented in this study are available on reasonable request from the corresponding author. The data are not publicly available due to privacy and ethical restrictions, and any data sharing will be subject to approval by the institutional ethics committee.

## Supplementary Materials

The following supporting information can be downloaded at https://www.mdpi.com/article/10.3390/jcm15124523/s1, Table S1: Information about missing data; Table S2: Odds ratios for spontaneous preterm delivery at <34 weeks of gestation calculated for each risk factor using multivariate analysis (complete-case analysis).

## Abbreviations

The following abbreviations are used in this manuscript:

AUC	area under the curve
CIs	confidence intervals
CL	cervical length
NPV	negative predictive value
ORs	odds ratios
PPV	positive predictive value
sPTB	spontaneous preterm birth
