# Supplementary material for: Longitudinal Ultrasound Evaluation of Cervical Length for Predicting Spontaneous Preterm Delivery Before 34 Weeks in Twin Gestations: A Retrospective Cohort Study
Source: J Clin Med. 2026 Jun 11;15(12):4523. doi: 10.3390/jcm15124523 (PMC13302035; doi:10.3390/jcm15124523)
Supplement: Supplementary file 1 [file jcm-15-04523-s001.zip › jcm-4314347-supplementary.pdf]

Supplementary Material

Table S1. Information about missing data

Variables	Number (%)
Maternal age	0 (0.0)
BMI on admission	0 (0.0)
Parity	0 (0.0)
Previous preterm birth	0 (0.0)
Conization	0 (0.0)
GDM/DM	0 (0.0)
HDP	0 (0.0)
Myoma	0 (0.0)
ART	0 (0.0)
Chorionicity	0 (0.0)
CL 18–21 weeks of gestation	0 (0.0)
CL 22–23 weeks of gestation	77 (36.3)
CL 24–25 weeks of gestation	59 (27.8)
CL 26–27 weeks of gestation	38 (17.9)
CL 28–29 weeks of gestation	29 (13.7)
CL 30–31 weeks of gestation	17 (8.0)
CL 32–33 weeks of gestation	28 (13.2)

BMI, body mass index; GDM, gestational diabetes mellitus; HDP, hypertensive disorders of pregnancy; ART, assisted reproductive technology

Table S2. Odds ratios for spontaneous preterm delivery at <34 weeks of gestation calculated for each risk factor using multivariate analysis (complete-case analysis).

	sPTB at <34 weeks (n=11)	Not sPTB at <34 weeks (n=70)	Crude OR (95% CI)	Adjusted OR (95% CI)
Model 1*				
High risk (n=34) (%)	29.4 (10/34)	70.6 (24/34)	19.17 (2.31–158.74)	17.35 (2.07–145.56)
Non-high risk (n=47) (%)	2.1 (1/47)	97.9 (46/47)	reference	reference
Model 2†				
High risk (n=34) (%)	23.5 (8/34)	76.5 (26/34)	4.51 (1.10–18.53)	3.97 (0.94–16.72)
Non-high risk (n=47) (%)	6.4 (3/47)	93.6 (44/47)	reference	reference
Model 3‡				
High risk (n=13) (%)	61.5 (8/13)	38.5 (5/13)	34.67 (6.94–173.22)	44.94 (7.45–271.19)
Non-high risk (n=68) (%)	4.4 (3/68)	95.6 (65/68)	reference	reference

sPTB, spontaneous preterm birth; OR, odds ratio; CI, confidence interval

*: High risk was defined as cervical length ≤20 mm at 22w0d–25w6d or cervical length ≤15 mm at 26w0d–33w6d, or a decrease in cervical length ≥10 mm in a measurement interval

†: High risk was defined as cervical length ≤20 mm at 22w0d-25w6d or cervical length ≤15 mm at 26w0d–33w6d

‡: High risk was defined as a decrease in cervical length ≥10 mm in a measurement interval
